# Role of Chrysophanol in Epithelial-Mesenchymal Transition in Oral Cancer Cell Lines via a Wnt-3-Dependent Pathway

**DOI:** 10.1155/2020/8373715

**Published:** 2020-09-15

**Authors:** Ping-Chen Chung, Po-Chun Hsieh, Chou-Chin Lan, Po-Chih Hsu, Min-Yi Sung, Ya-Hsuan Lin, I.-Shiang Tzeng, Valeria Chiu, Ching-Feng Cheng, Chan-Yen Kuo

**Affiliations:** ^1^Department of Anesthesia, Chang Bing Show Chwan Memorial Hospital, Changhua, Taiwan; ^2^Department of Chinese Medicine, Taipei Tzu Chi Hospital, Buddhist Tzu Chi Medical Foundation, New Taipei City, Taiwan; ^3^Division of Pulmonary Medicine, Taipei Tzu Chi Hospital, Buddhist Tzu Chi Medical Foundation, New Taipei City, Taiwan; ^4^Department of Dentistry, Taipei Tzu Chi Hospital, Buddhist Tzu Chi Medical Foundation, New Taipei City, Taiwan; ^5^Department of Research, Taipei Tzu Chi Hospital, Buddhist Tzu Chi Medical Foundation, New Taipei City, Taiwan; ^6^Division of Physical Medicine and Rehabilitation, Taipei Tzu Chi Hospital, Buddhist Tzu Chi Medical Foundation, New Taipei City, Taiwan; ^7^Department of Pediatrics, Taipei Tzu Chi Hospital, Buddhist Tzu Chi Medical Foundation, Taipei, Taiwan; ^8^Institute of Biomedical Sciences, Academia Sinica, Taipei, Taiwan; ^9^Department of Pediatrics, Tzu Chi University, Hualien, Taiwan

## Abstract

Oral cancer belongs to the group of head and neck cancers. If not diagnosed or treated early, it can be life threatening. Epithelial-mesenchymal transition (EMT) plays an important role in tumor formation and progression. An increase in the presence of the EMT phenotype causes tumor cell proliferation, migration, invasion, and poor prognosis. Therefore, attenuating carcinogenesis via EMT inhibition is a good strategy. Herein, we will determine the pharmacological effects of chrysophanol on the EMT in FaDu cells. To analyze EMT, we detected the expression EMT markers, including *α*-SMA, *β*-catenin, vimentin, N-cadherin, E-cadherin, phospho-GSK-3*β*, and nuclear translocations of p65 and *β*-catenin by western blotting. Additionally, accumulating evidence indicates that reactive oxygen species (ROS) mediate EMT. Our results showed that the level of ROS was significantly increased after chrysophanol treatment. We further speculated that chrysophanol-mediated EMT and metastasis are involved in the Wnt-3-dependent signaling pathway. The inhibition of the EMT phenotype and metastasis and accumulation of ROS caused by chrysophanol was reversed by treatment with the Wnt-3 agonist Bml 284. Therefore, our findings indicated that chrysophanol altered EMT formation, ROS accumulation, and metastasis via the Wnt-3-dependent signaling pathway.

## 1. Introduction

Oral cancer is a serious health care problem around the world [1]. It includes oral squamous cell carcinoma (OSCC) and head and neck squamous cell carcinoma (HNSCC) [2]. It has been reported that OSCC is one of the most common cancers influencing the head and neck region, accounting for about 90% of oral cancers and early diagnosis and treatment increase the survival rate [3]. Importantly, epithelial-mesenchymal transition (EMT) is emerging as a hallmark in OSCC and could be proposed as a promising strategy to improve the outcomes of patients with OSCC [4]. EMT was characterized by the loss of epithelial markers and the gain of mesenchymal markers, such as vimentin and nuclear translocation of *β*-catenin [5], as well as upregulation of *α*-smooth muscle cell actin (*α*-SMA), fibronectin, and cadhern-11 [5, 6]. Therefore, suppressing the phenomena of EMT is considered an effective option to ameliorate oral cancer. Currently, exposure of oral cancer patients treated with chemoradiotherapy to related acute or late toxicities may cause oral complications that may not be treated and recognized. Acute toxic effects include mucositis, salivary gland dysfunction, and pain. On the other hand, the late toxic effects of chemoradiotherapy include dysphagia, dehydration, and dysgeusia [1]. It has been reported that radiation therapy can lead to xerostomia, rampant dental caries secondary to dry mouth, trismus, and osteoradionecrosis [2]. Moreover, Ginsenoside Rg3, isolated from the roots of *Panax ginseng*, has an anticarcinogenic effect on human oral squamous carcinoma cells via EMT inhibition, miR-221 downregulation, TIMP3 upregulation, and then PI3K/AKT inactivation and the MAPK/ERK pathways [3]. Therefore, it is necessary to identify other potential natural products that can suppress EMT to ameliorate further oral cancer development.

Moreover, Li et al. suggested that chemokine (C-C motif) ligand 2 (CCL2) is critical for the regulation of endogenous ROS production in OSCC SCC-9 and CAL-27 cells [4]. Accumulating evidence has been reported that ROS is a key regulator in the development of oral cancer [5–7]. On the other hand, Liu et al. reported that melatonin reduced proliferation and apoptosis resistance in oral cancer cells by inactivating ROS-dependent Akt signaling [8]. Therefore, ROS may be considered as a therapeutic target in the progression of oral cancer. Dahuang, the roots of *Rheum palmatum*, a well-known traditional Chinese herb used for chronic kidney disease and liver disease [9, 10]. Chrysophanol, an anthraquinone derivative isolated from the rhizomes of Rheum palmatum, has been reported to inhibit proliferation and induces apoptosis through the NF-*κ*B/cyclin D1 and NF-*κ*B/Bcl-2 signaling cascade in breast cancer cell lines [11]. However, the pharmacologic effects of chrysophanol and its underlying mechanisms in oral cancer cell lines are still unknown. The aim of this study was to investigate the effects of chrysophanol on the oral cancer cell line FaDu and its mechanisms of action. Additionally, accumulating evidence showed that ROS regulates the EMT process in carcinogenesis [12, 13]. Therefore, it is important to identify candidate compounds to prevent the progression of EMT in oral cancer cell lines via overproduction of ROS. In our previous study, chrysophanol was found to regulate cell death, metastasis, ROS production, and ferroptosis in oral cancer cell lines [14]; however, the key mechanism in EMT formation after chrysophanol treatment is still unclear. Thus, the aim of this study is to speculate on the role of chrysophanol in EMT in oral cancer cell lines via the Wnt-3 signaling pathway.

## 2. Materials and Methods

### 2.1. Reagents

Chrysophanol, glutathione, and NAC were supplied by Sigma (St. Louis, MO, USA). Bml 284 (Enzo Life Sciences, Inc., NY, USA).

### 2.2. Antibodies

Antibodies for western blot analyses and the final working dilutions were as follows: rabbit polyclonal antibodies to *α*-SMA (dilution 1 : 1000; ABclonal, MA, USA), *β*-catenin (dilution 1 : 1000; ABclonal, MA, USA), vimentin (dilution 1 : 500; Santa Cruz, TX, USA), N-cadherin (dilution 1 : 1000; ABclonal, MA, USA), E-cadherin (dilution 1 : 1000; ABclonal, MA, USA), Wnt-3 (dilution 1 : 1000; ABclonal, MA, USA), p-GSK3*β* (dilution 1 : 1000; ABclonal, MA, USA), GSK3*β* (dilution 1 : 1000; ABclonal, MA, USA), *β*-actin (dilution 1 : 1000, Santa Cruz, TX, USA).

p65 (dilution 1 : 1000; GeneTex, CA, USA), and fibrillin (dilution 1 : 1000, ABclonal, MA, USA). All HRP-conjugated secondary antibodies were purchased from Jackson ImmunoResearch Inc. (PA, USA).

### 2.3. Cell Culture

The human oral cell line FaDu was purchased from the ATCC (Manassas, VA). Cells were analyzed for mycoplasma contamination and tested negative. Cells were cultured in Dulbecco's Modified Eagle Medium containing 10% Fetal Bovine Serum and 1% Penicillin/Streptomycin and incubated in a 5% CO_2_ atmosphere at 37°C.

### 2.4. Western Blotting

Cells were collected, resuspended in ice-cold relaxation buffer (20 mM Tris-HCl (pH 7.4), 150 mM NaCl, 1 mM EGTA, 1 mM NaF, 2 mM Na_3_VO_4_, 1 mM phenylmethylsulphonyl fluoride, a 1% dilution of Sigma protease inhibitor cocktail, and 1% Triton X-100), and boiled in (sodium dodecyl sulfate) SDS sample buffer. Equal amounts of protein were separated on SDS-PAGE and then transferred to polyvinylidene difluoride membranes (Millipore, Bedford, MA). The membrane was blocked with 1% BSA and incubated with specific primary antibodies overnight. The appropriate HRP-conjugated secondary antibodies (1 : 5000; Jackson Immunoresearch) were incubated for 1 h at room temperature, and expression of protein was detected using an ECL kit (Millipore, Temecula, CA).

### 2.5. Nuclear Fraction Extraction

The nuclear fraction was extracted from FaDu cells. The cells were collected and resuspended in a hypotonic buffer (10 mM HEPES, pH7.9; 10 mM KCl; 1.5 mM MgCl_2_; 0.2 mM PMSF; 20 *μ*g/ml aprotinin; 0.5 mM DTT; and 0.5% NP-40) on ice for 15 minutes. After centrifuging at 6,000 × *g* for 15 minutes at 4°C, the pellet was collected and then washed with basal buffer (hypotonic buffer without 0.5% NP-40). After centrifuging again at 6,000 × *g* for 15 minutes at 4°C, the pellet was collected and resuspended in a hypertonic buffer (20 mM HEPES, pH 7.9; 400 mM KCl; 1.5 mM MgCl_2_; 0.2 mM PMSF; 20 *μ*g/ml aprotinin; 0.5 mM DTT; 0.2 mM EDTA; 10% glycerol) at room temperature for 30 minutes. After centrifuging at 10,000 × *g* for 30 minutes at 4°C, the nuclear fraction contained in the supernatant was collected.

### 2.6. Adhesion Analysis

The adhesion analysis was used according to previous study [15] with some modification. Briefly, cells (1 × 10^5^) were seed in the 48-well plate with serum-free medium (DMEM containing 0.5% BSA, 2 mM CaCl_2_, and 2 mM MgCl_2_). After treatment with different conditions, the ability of adhesion was measured according to the manufacturer's instructions (CytoSelect™ Tumor-Endothelium Adhesion Assay, Cell Biolabs, Inc., San Diego, CA, USA). Finally, the fluorescence was read with a fluorescence plate reader at 485/530 nm (Synergy HT, BioTek, VT, USA).

### 2.7. Transwell Metastasis Assay

A 24-well transwell plate (8-*μ*m pore size, Corning, NY, USA) was used to measure the metastasis ability of the FaDu cells in three conditions. First, FaDu cells were seeded (1 × 10^5^) in the upper well. After incubation for 48 hours, cells were treated with chrysophanol or Bml 284 for 24 hours in serum-free medium supplemented with 5% bovine serum albumin (BIONOVAS, Toronto, Canada). Then, a noncoated membrane in serum-free medium supplemented with 5% bovine serum albumin (BIONOVAS, Toronto, Canada) was added to the lower well. After 24 hours, the migratory cells on the underside of the membrane were stained with 0.1% crystal violet for 5 minutes and washed with H_2_O; the membrane was analyzed using a Nikon E400 phase-contrast microscope (Nikon, Tokyo, Japan). The crystal violet was destained with methanol for 15 minutes, and the absorbance values were measured at OD570. All measured values were detected by Synergy HT (BioTek, VT, USA).

### 2.8. Statistical Analysis

Continuous data were expressed as mean ± standard error of the mean. Statistical differences in means among groups were determined by one-way or a two-way analysis of variance with a Bonferroni post hoc test for continuous variables. *P* values <0.05 were considered to indicate statistically significant differences.

## 3. Results

### 3.1. Chrysophanol Alleviated EMT Phenotype Formation via a Wnt-3-Dependent Signaling Pathway

**Figure 1:**
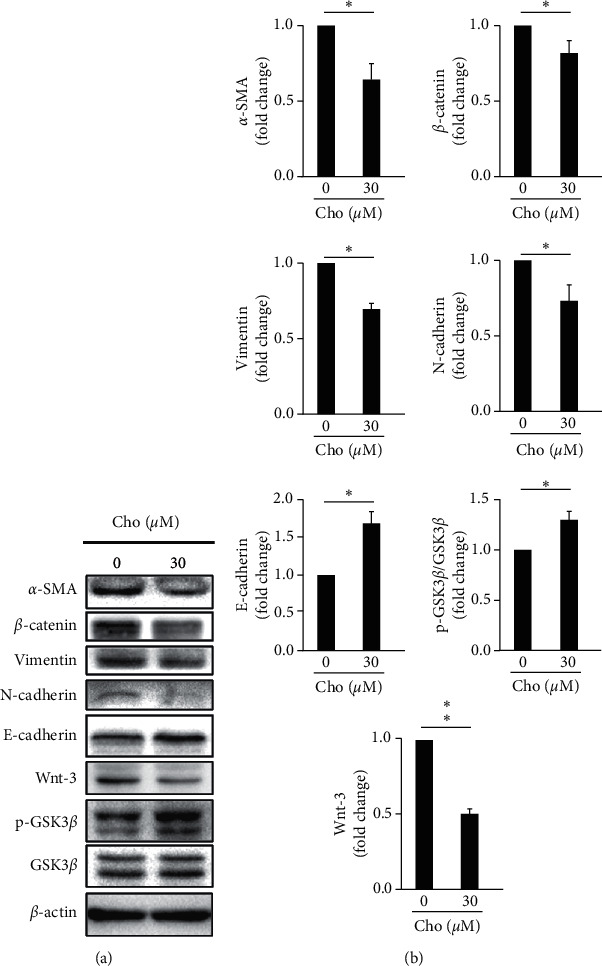
The effect of chrysophanol on the expression of EMT markers, Wnt-3, pGSK3*β*, and GSK3*β*. (a) FaDu cells were treated with either the control (0 *μ*M) or the indicated concentration (30 *μ*M) of chrysophanol (Cho) for 24 h. Cells were then harvested and the proteins were separated by SDS-PAGE, followed by immunoblotting with the indicated antibodies. *β*-actin was used as an internal control. (b) Densitometric analysis of all samples was normalized against the level of total protein. The images are representative of the results of three independent experiments. All data are presented as the mean ± SD. ^*∗*^*P* < 0.05.

**Figure 2:**
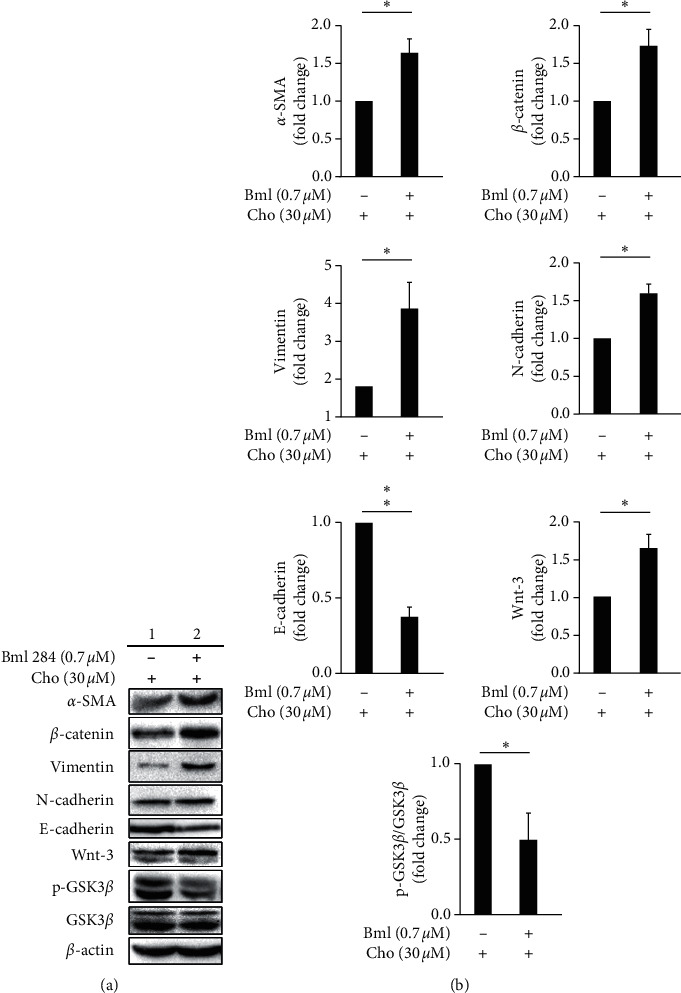
The effect of the Wnt-3/pGSK3*β* activator, Bml 284 on the expression of EMT markers, Wnt-3, pGSK3*β*, and GSK3*β*. (a) FaDu cells were treated with either the control (0 *μ*M, column 1) or the indicated concentration (0.7 *μ*M) of Bml 284 (column 2) in the presence of 30 *μ*M chrysophanol (Cho) for 24 h. Cells were then harvested and the proteins were separated by SDS-PAGE, followed by immunoblotting with the indicated antibodies. *β*-actin was used as an internal control. (b) Densitometric analysis of all samples normalized against the level of total protein. The images are representative of the results of three independent experiments. All data are presented as the mean ± SD. ^*∗*^*P* < 0.05. ^*∗∗*^*P* < 0.01.

To investigate the effect of chrysophanol on EMT formation, we detected the expression of EMT markers including *α*-SMA, *β*-catenin, vimentin, N-cadherin, E-cadherin, and phospho-GSK-3*β* with and without chrysophanol by western blotting. The results indicated that *α*-SMA, *β*-catenin, N-cadherin, and vimentin decreased, but E-cadherin and phospho-GSK-3*β* increased after chrysophanol treatment, as shown in Figures 1(a) and 1(b). On the other hand, Wnt-3 was downregulated after 30 *μ*M chrysophanol treatment (Figure 1(a), lower panel; 1(b)). To further verify whether chrysophanol attenuated EMT in the Wnt-3-dependent signaling pathway, we detected the expression of *α*-SMA, *β*-catenin, vimentin, N-cadherin, E-cadherin, and phospho-GSK-3*β* in the presence and absence of the Wnt agonist Bml 284 (0.7 *μ*M) after treatment with chrysophanol. The results showed that the decrease in *α*-SMA, *β*-catenin, N-cadherin, and vimentin and increase in E-cadherin and phospho-GSK-3*β* were reversed in the presence of Bml 284 (Figure 2).

### 3.2. Chrysophanol-Mediated Nuclear Translocation of p65 and *β*-Catenin in EMT Phenotype Formation via the Wnt-3-Dependent Signaling Pathway

**Figure 3:**
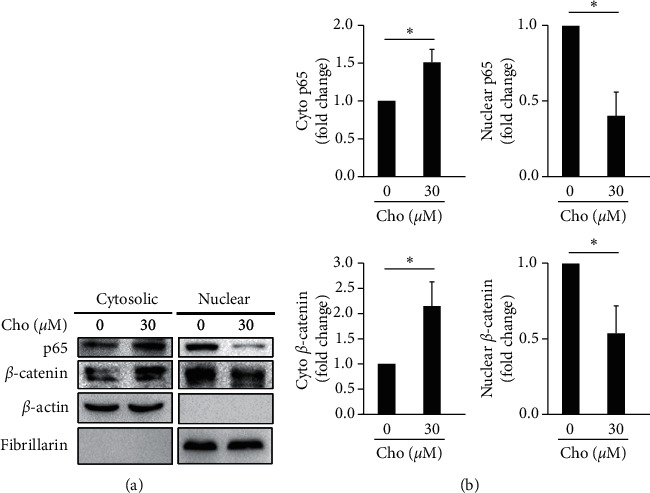
Chrysophanol attenuated NF-*κ*B and *β*-catenin nuclear translocation. (a) FaDu cells cultured and treated as above were collected, and cytosolic and nuclear fractions were isolated as described in Materials and Methods. Western blot analysis was performed to detect the subcellular localization of p65 and *β*-catenin using an antibody against the NF-*κ*B subunit p65. *β*-actin was used as a cytosolic marker and fibrillarin served as a nuclear marker. (b) Densitometric analysis of all samples normalized against the level of total protein. The images are representative of the results of three independent experiments. All data are presented as the mean ± SD. ^*∗*^*P* < 0.05. Cho indicated as chrysophanol.

**Figure 4:**
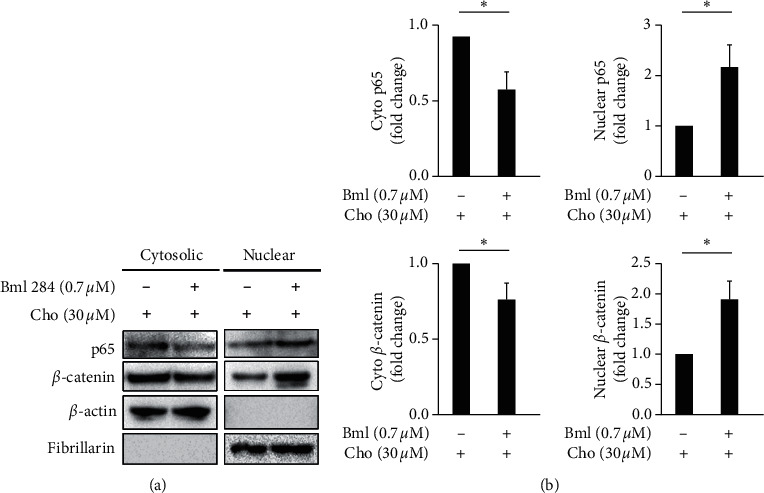
Bml 284 reversed chrysophanol attenuated NF-*κ*B and *β*-catenin nuclear translocation. (a) FaDu cells cultured and treated as above were collected, and cytosolic and nuclear fractions were isolated as described in Materials and Methods. Western blot analysis was performed to detect the subcellular localization of p65 and *β*-catenin using an antibody against the NF-*κ*B subunit p65. *β*-actin was used as a cytosolic marker, and fibrillarin served as a nuclear marker. (b) Densitometric analysis of all samples normalized against the level of total protein. The images are representative of the results of three independent experiments. All data are presented as the mean ± SD. ^*∗*^*P* < 0.05. Cho indicates chrysophanol.

In our previous study, chrysophanol was found to regulate cell death, metastasis, ROS production, and ferroptosis in oral cancer cell lines [14]; however, the molecular circuitry mediated by chrysophanol in the progression of EMT is poorly understood at this time. Accumulating evidence indicates that NF-*κ*B/p65 nuclear translocation is one of the key regulators of EMT in breast cancer and pancreatic ductal adenocarcinoma [16, 17] and that another is *β*-catenin in colorectal and prostate cancer [18, 19]. By using western blotting, we showed that chrysophanol attenuated EMT formation via inhibition of p65 and *β*-catenin nuclear translocation (Figure 3) and that this inhibition was reversed after treatment with a Wnt agonist, Bml 284 (0.7 *μ*M) (Figure 4). As such, we propose that the regulatory mechanisms of the EMT pathway related to Wnt-3/*β*-catenin and p65 played a critical role in the development of oral cancer and thus in the inhibition caused by chrysophanol.

### 3.3. Chrysophanol Alleviates Metastasis and Adhesion via a Wnt-3-Dependent Signaling Pathway

**Figure 5:**
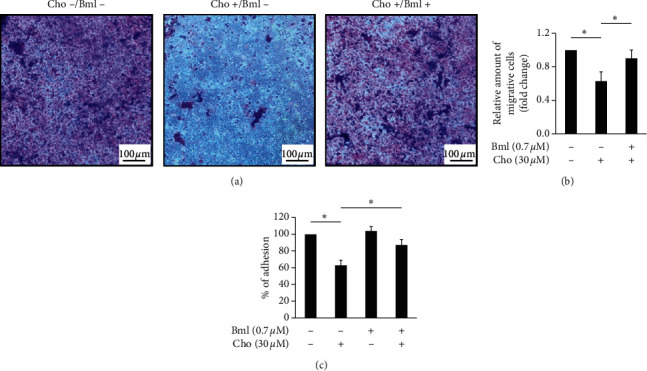
Bml 284 attenuated chrysophanol-induced migration and adhesion inhibition. (a) Chrysophanol-treated FaDu cells were incubated with (Cho+/Bml+, lower panel) or without (Cho+/Bml−, middle panel) 0.7 *μ*M Bml 284 for 24 h. Macroscopic observation of transwell chambers is shown on the top. (b) Relative amounts of migrated cells are presented as the extent of crystal violet staining measured at OD570 and shown in the bottom panels. (c) Cell adhesion assay. The adherent cells were stained with CytoSelect™ and determined by measuring the fluorescence at 485/530 nm. All data are presented as the mean ± SD. ^*∗*^*P* < 0.05.

Cell metastasis is a hallmark of cancer that is responsible for the greatest number of cancer-related deaths [20]. Therefore, the role of FaDu cells in oral cancer cell metastasis was investigated with transwell assays. As shown in Figures 5(a) and 5(b), after treatment with chrysophanol, cell migration was decreased significantly compared with the control (without chrysophanol treatment). On the other hand, the inhibition of metastasis caused by chrysophanol was reversed after treatment with Bml 284 (0.7 *μ*M) (Figures 5(a) and 5(b)). We also detected the adhesion ability which altered after treatment with chrysophanol via Wnt-3-dependent pathway. Results showed that cell adhesion was decreased significantly compared with the control (without chrysophanol treatment) but was reversed after treatment with Bml 284 (0.7 *μ*M) (Figure 5(c)). These results indicated that chrysophanol decreased metastasis via a Wnt-3-dependent signaling pathway.

## 4. Discussion

The poor prognosis of OSCC is mainly caused by its high recurrence and metastasis rates and remains a significant medical challenge, regardless of advances in diagnostic and therapeutic procedures [21, 22]. Accumulating evidence suggests that EMT plays an important role during oral cancer invasion and metastasis [23, 24]. Additionally, metastasis has been considered as a key element of poor prognosis [25–30]. Therefore, it is well established that targeting EMT/metastasis in oral carcinogenesis, and we have provided an update on an EMT/metastasis inhibitor that has emerged as a promising candidate for therapeutic interventions against oral cancer in clinical studies. In the present study, western blotting results showed that chrysophanol decreased the expression of EMT markers (Figure 1). We further showed that chrysophanol demonstrates its anti-invasive effect through modulation of the EMT process (Figures 1, 3, and 5). To further confirm whether chrysophanol-induced EMT alteration is via Wnt-3-dependent signaling pathway, we detected the expressions of *α*-SMA, *β*-catenin, vimentin, N-cadherin, E-cadherin, Wnt-3, and phospho-GSK-3*β* in the presence of Bml 284 (0.7 *μ*M) (Supplemental figure 1(a)). Results showed that the expression of indicated proteins was not altered in presence of Wnt-3 agonist, Bml 284 (Supplemental figure 1(a)), as was found in p65 and *β*-catenin nuclear translocation (Supplemental figure 1(b)). Additionally, Bml284 is a potent cell-permeable Wnt signaling activator by inducing *β*-catenin and TCF-dependent transcriptional activity [31]. Since the regulation is at the transcriptional level, Bml284 is used to validate the effects on the Wnt signaling pathway. In the present study, Western blot analysis results indicate that chrysophanol alters Wnt-3 expression and EMT formation of FaDu cells, which is reversed by Bml284 induction, recognizing the effect on Wnt-3 signaling cascade. Overall, we clarified that chrysophanol-induced EMT alteration is via Wnt-3-dependent signaling pathway in FaDu cells.

Wnt-3 and GSK-3*β* were significantly upregulated in OSCC in comparison with control tissues [32]. Uraguchi et al. reported that Wnt-3 was localized to carcinoma cells at the invasive front but was not expressed in normal oral tissues, and that of CTNNB1 was localized to the nucleus and diffusively to the cytoplasm [33]. On the other hand, Ishida et al. reported that the nuclear localization of *β*-catenin was detected in oral leukoplakia with dysplasia but not in epithelial cells without dysplasia [34]. Jamieson et al. proposed that targeting the *β*-catenin nuclear import pathway may provide an opportunity to identify specific drug targets via inhibition of the *β*-catenin nuclear function or translocation in cancer progression [35]. Our results showed that chrysophanol inhibited the nuclear localization of *β*-catenin in FaDu cells (Figure 3), which is in agreement with previous studies [35, 36]. Furthermore, we further showed that the phenotype of EMT was reversed by Bml 284, which was consistent with previous studies [37, 38]. On the other hand, substantial evidence has shown that Wnt-3 is a key regulator of cell proliferation and metastasis in colorectal cancer, gastric cancer development, and malignant melanoma [39–41]. In the present study, our results showed that chrysophanol inhibits metastasis by decreasing Wnt-3 upregulation (Figure 5), which is in agreement with previous studies [42–44]. Therefore, the inhibition of metastasis via downregulation of Wnt-3 will be a valuable strategy in cancer treatment.

**Figure 6:**
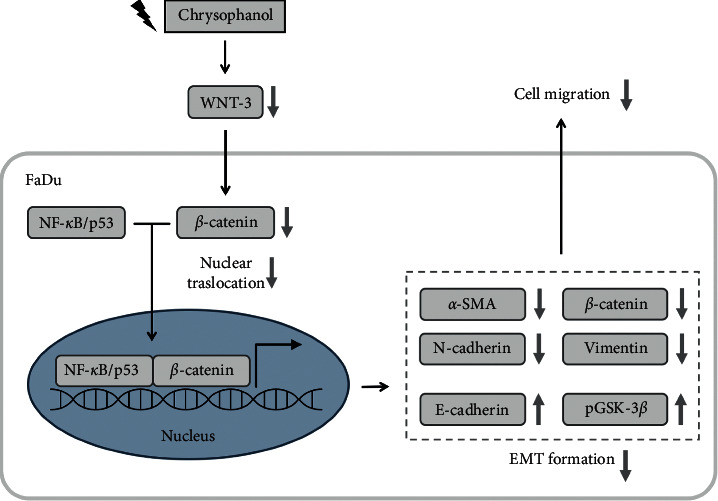
A summary diagram outlines the working mechanisms of oridonin in FaDu cells. (a) Chrysophanol caused ROS accumulation. (b) Chrysophanol downregulated the expressions of Wnt-3 and nuclear translocations of NF-*κ*B/p65 or *β*-catenin. On the other hand, chrysophanol also attenuated EMT formation and cell migration.

In conclusion, chrysophanol significantly inhibited EMT formation and metastasis via a Wnt-3-dependent signaling pathway. On the other hand, chrysophanol caused ROS accumulation via a Wnt-3-dependent signaling pathway (Figure 6). Our pharmacological findings support Wnt-3 as a therapeutic target in oral cancer development and metastasis.

## Data Availability

The original data used to support the findings of this study are included in the article.

## Supplementary Materials

Supplementary Materials Supplemental Figure 1: the effect of Bml on EMT markers, Wnt-3, pGSK3*β*, GSK3*β*, and nuclear translocation of NF-*κ*B and *β*-catenin. (a) FaDu cells were treated with either the control (0 *μ*M) or the indicated concentration (0.7 *μ*M) of Bml 284 for 24 h. Cells were then harvested and the proteins separated by SDS-PAGE, followed by immunoblotting with the indicated antibodies. *β*-actin was used as an internal control. (b) FaDu cells cultured and treated as above were collected, and cytosolic and nuclear fractions were isolated as described in Materials and Methods. Western blot analysis was performed to detect the subcellular localization of p65 and *β*-catenin using an antibody against the NF-*κ*B subunit p65. *β*-actin was used as a cytosolic marker, and fibrillarin served as a nuclear marker.

## References

[1] Haddad R. I., Shin D. M. (2008). Recent advances in head and neck cancer. *New England Journal of Medicine*.

[2] Epstein J. B., Thariat J., Bensadoun R.-J. (2012). Oral complications of cancer and cancer therapy: from cancer treatment to survivorship. *CA: A Cancer Journal for Clinicians*.

[3] Cheng Z., Xing D. (2019). Ginsenoside Rg3 inhibits growth and epithelial-mesenchymal transition of human oral squamous carcinoma cells by down-regulating miR-221. *European Journal of Pharmacology*.

[4] Li X., Xu Q., Wu Y. (2014). A CCL2/ROS autoregulation loop is critical for cancer-associated fibroblasts-enhanced tumor growth of oral squamous cell carcinoma. *Carcinogenesis*.

[5] Lin C. C., Yang J. S., Chen J. T. (2007). Berberine induces apoptosis in human HSC-3 oral cancer cells via simultaneous activation of the death receptor-mediated and mitochondrial pathway. *Anticancer Research*.

[6] Kang J. W., Kim J. H., Song K., Kim S. H., Yoon J.-H., Kim K.-S. (2010). Kaempferol and quercetin, components of Ginkgo Biloba extract (EGb 761), induce caspase-3-dependent apoptosis in oral cavity cancer cells. *Phytotherapy Research*.

[7] NavaneethaKrishnan S., Rosales J. L., Lee K. Y. (2019). ROS-mediated cancer cell killing through dietary phytochemicals. *Oxidative Medicine and Cellular Longevity*.

[8] Liu R., Wang H. L., Deng M. J. (2018). Melatonin inhibits reactive oxygen species-driven proliferation, epithelial-mesenchymal transition, and vasculogenic mimicry in oral cancer. *Oxidative Medicine and Cellular Longevity*.

[9] Fang J., Sun X., Xue B., Fang N., Zhou M. (2017). Dahuang Zexie decoction protects against high-fat diet-induced NAFLD by modulating gut microbiota-mediated toll-like receptor 4 signaling activation and loss of intestinal barrier. *Evidence-Based Complementary and Alternative Medicine*.

[10] Tu Y., Sun W., Wan Y.-G. (2014). Dahuang Fuzi decoction ameliorates tubular epithelial apoptosis and renal damage via inhibiting TGF-*β*1-JNK signaling pathway activation in vivo. *Journal of Ethnopharmacology*.

[11] Ren L., Li Z., Dai C. (2018). Chrysophanol inhibits proliferation and induces apoptosis through NF-kappaB/cyclin D1 and NF-kappaB/Bcl-2 signaling cascade in breast cancer cell lines. *Molecular Medicine Reports*.

[12] Jiang J., Wang K., Chen Y., Chen H., Nice E. C., Huang C. (2017). Redox regulation in tumor cell epithelial-mesenchymal transition: molecular basis and therapeutic strategy. *Signal Transduction and Targeted Therapy*.

[13] Cichon M. A., Radisky D. C. (2014). ROS-induced epithelial-mesenchymal transition in mammary epithelial cells is mediated by NF-*κ*B-dependent activation of Snail. *Oncotarget*.

[14] Hsu P.-C., Cheng C.-F., Hsieh P.-C., Chen Y.-H., Kuo C.-Y., Sytwu H.-K. (2020). Chrysophanol regulates cell death, metastasis, and reactive oxygen species production in oral cancer cell lines. *Evidence-Based Complementary and Alternative Medicine*.

[15] Kopf S., Viola K., Atanasov A. G. (2013). In vitro characterisation of the anti-intravasative properties of the marine product heteronemin. *Archives of Toxicology*.

[16] Pires B. R., Mencalha A. L., Ferreira G. M. (2017). NF-kappaB is involved in the regulation of EMT genes in breast cancer cells. *PLoS One*.

[17] Gao S., Sun Y., Zhang X. (2016). IGFBP2 activates the NF-*κ*B pathway to drive epithelial-mesenchymal transition and invasive character in pancreatic ductal adenocarcinoma. *Cancer Research*.

[18] Kim W. K., Kwon Y., Jang M. (2019). Beta-catenin activation down-regulates cell-cell junction-related genes and induces epithelial-to-mesenchymal transition in colorectal cancers. *Scientific Reports*.

[19] Luo Y., Li M., Zuo X. (2018). Betacatenin nuclear translocation induced by HIF1alpha overexpression leads to the radioresistance of prostate cancer. *International Journal of Oncology*.

[20] Fares J., Fares M. Y., Khachfe H. H., Salhab H. A., Fares Y. (2020). Molecular principles of metastasis: a hallmark of cancer revisited. *Signal Transduction and Targeted Therapy*.

[21] Lazarevic M., Milosevic M., Jelovac D. (2020). Marked epithelial to mesenchymal transition in surgical margins of oral cancer-an in vitro study. *Oncology Letters*.

[22] Feller L. L., Khammissa R. R., Kramer B. B., Lemmer J. J. (2013). Oral squamous cell carcinoma in relation to field precancerisation: pathobiology. *Cancer Cell International*.

[23] Patil S., Rao R. S., Ganavi B. S. (2015). Mesenchymal-epithelial transition in oral cancer. *Journal of International Oral Health*.

[24] Krisanaprakornkit S., Iamaroon A. (2012). Epithelial-mesenchymal transition in oral squamous cell carcinoma. *International Scholarly Research Notices*.

[25] Bernardi M. A., Logullo A. F., Pasini F. S. (2012). Prognostic significance of CD24 and claudin-7 immunoexpression in ductal invasive breast cancer. *Oncology Reports*.

[26] Chao Y.-C., Pan S.-H., Yang S.-C. (2009). Claudin-1 is a metastasis suppressor and correlates with clinical outcome in lung adenocarcinoma. *American Journal of Respiratory and Critical Care Medicine*.

[27] DeVita V. T., Young R. C., Canellos G. P. (1975). Combination versus single agent chemotherapy: a review of the basis for selection of drug treatment of cancer. *Cancer*.

[28] Folkman J., Shing Y. (1992). Angiogenesis. *The Journal of Biological Chemistry*.

[29] Ono M., Torisu H., Fukushi J., Nishie A., Kuwano M. (1999). Biological implications of macrophage infiltration in human tumor angiogenesis. *Cancer Chemotherapy and Pharmacology*.

[30] Wittekind C., Neid M. (2005). Cancer invasion and metastasis. *Oncology*.

[31] Liu J., Wu X., Mitchell B., Kintner C., Ding S., Schultz P. G. (2005). A small-molecule agonist of the Wnt signaling pathway. *Angewandte Chemie International Edition*.

[32] Andrade Filho P. A., Letra A., Cramer A. (2011). Insights from studies with oral cleft genes suggest associations between WNT-pathway genes and risk of oral cancer. *Journal of Dental Research*.

[33] Uraguchi M., Morikawa M., Shirakawa M., Sanada K., Imai K. (2004). Activation of WNT family expression and signaling in squamous cell carcinomas of the oral cavity. *Journal of Dental Research*.

[34] Ishida K., Ito S., Wada N. (2007). Nuclear localization of beta-catenin involved in precancerous change in oral leukoplakia. *Molecular Cancer*.

[35] Jamieson C., Sharma M., Henderson B. R. (2014). Targeting the *β*-catenin nuclear transport pathway in cancer. *Seminars in Cancer Biology*.

[36] Reyes M., Peña-Oyarzun D., Maturana A., Torres V. A. (2019). Nuclear localization of *β*-catenin and expression of target genes are associated with increased Wnt secretion in oral dysplasia. *Oral Oncology*.

[37] Jiang Y., Wang W., Wu X., Shi J. (2020). Pizotifen inhibits the proliferation and invasion of gastric cancer cells. *Experimental and Therapeutic Medicine*.

[38] Zeng X., Zhang Y., Xu H., Zhang T., Xue Y., An R. (2018). Secreted frizzled related protein 2 modulates epithelial-mesenchymal transition and stemness via wnt/*β*-catenin signaling in choriocarcinoma. *Cellular Physiology and Biochemistry*.

[39] Nie X., Xia F., Liu Y. (2019). Downregulation of Wnt3 suppresses colorectal cancer development through inhibiting cell proliferation and migration. *Frontiers in Pharmacology*.

[40] Wang Y.-D., Nie X., Wu R.-B. (2016). Downregulation of human Wnt3 in gastric cancer suppresses cell proliferation and induces apoptosis. *OncoTargets and Therapy*.

[41] Sinnberg T., Levesque M. P., Krochmann J. (2018). Wnt-signaling enhances neural crest migration of melanoma cells and induces an invasive phenotype. *Molecular Cancer*.

[42] DiMeo T. A., Anderson K., Phadke P. (2009). A novel lung metastasis signature links Wnt signaling with cancer cell self-renewal and epithelial-mesenchymal transition in basal-like breast cancer. *Cancer Research*.

[43] Gao Y., Lin W., Zhou S., Shi G., He J., Chen Y. (2018). Treatment of Rosacea using acupuncture for improving the local skin microcirculation: a case report. *Medicine (Baltimore)*.

[44] Jung Y.-S., Park J.-I. (2020). Wnt signaling in cancer: therapeutic targeting of Wnt signaling beyond *β*-catenin and the destruction complex. *Experimental & Molecular Medicine*.

