# Supplementary material for: Role of Chrysophanol in Epithelial-Mesenchymal Transition in Oral Cancer Cell Lines via a Wnt-3-Dependent Pathway
Source: Evid Based Complement Alternat Med. 2020 Sep 15;2020:8373715. doi: 10.1155/2020/8373715 (PMC7512067; doi:10.1155/2020/8373715)

**Supplemental figure 1. Effect of Bml on EMT markers, Wnt-3, pGSK3β, GSK3β, and nuclear translocation of NF-κB and β-catenin.** (A) FaDu cells were treated with either the control (0 μM) or the indicated concentration (0.7 μM) of Bml 284 for 24 h. Cells were then harvested and the proteins separated by SDS-PAGE, followed by immunoblotting with the indicated antibodies. β-actin was used as an internal control. (B) FaDu cells cultured and treated as above were collected, and cytosolic and nuclear fractions were isolated as described in Materials and methods. Western blot analysis was performed to detect the subcellular localization of p65 and β-catenin using an antibody against the NF-κB subunit p65. β-actin was used as a cytosolic marker, and fibrillarin served as a nuclear marker.


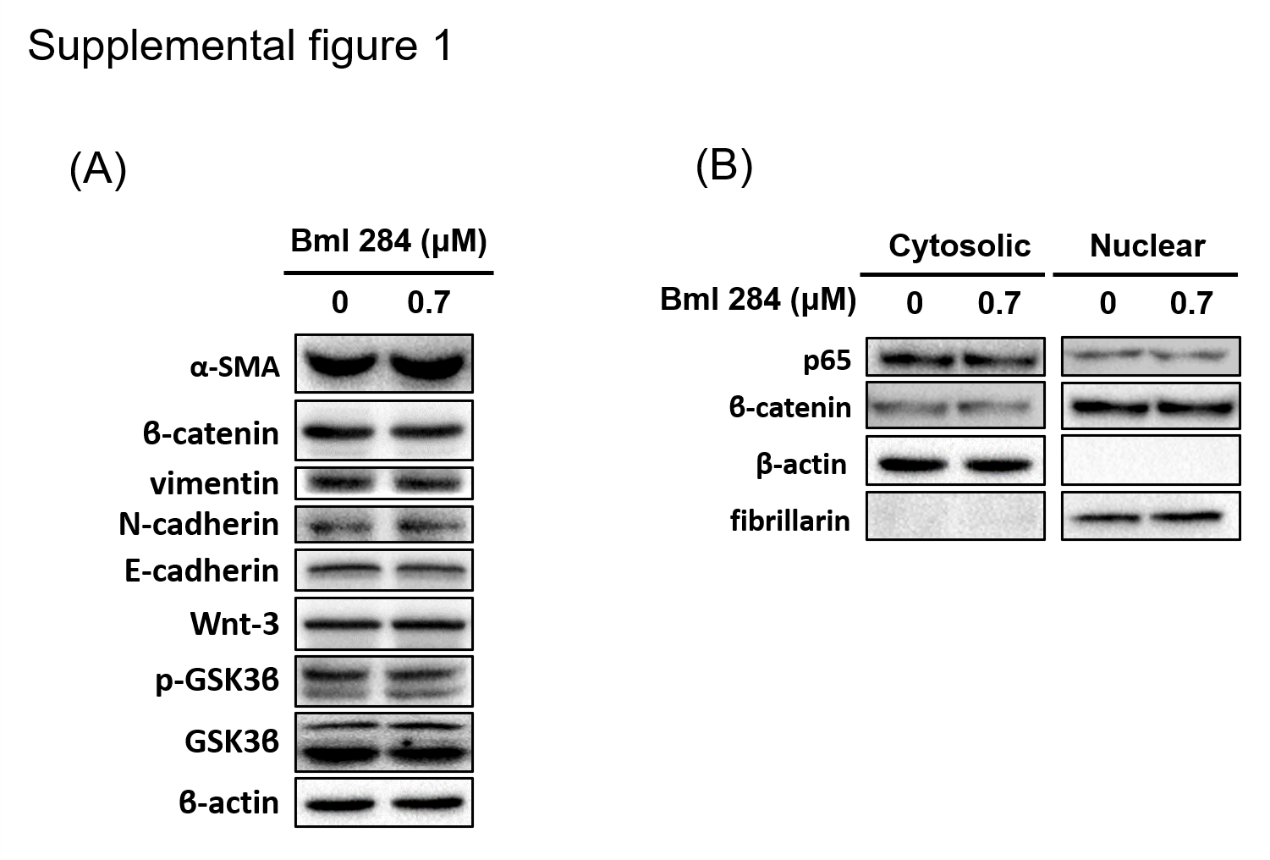

Supplement: Supplementary Materials — Supplemental Figure 1: the effect of Bml on EMT markers, Wnt-3, pGSK3β, GSK3β, and nuclear translocation of NF-κB and β-catenin. (a) FaDu cells were treated with either the control (0 μM) or the indicated concentration (0.7 μM) of Bml 284 for 24 h. Cells were then harvested and the proteins separated by SDS-PAGE, followed by immunoblotting with the indicated antibodies. β-actin was used as an internal control. (b) FaDu cells cultured and treated as above were collected, and cytosolic and nuclear fractions were isolated as described in Materials and Methods. Western blot analysis was performed to detect the subcellular localization of p65 and β-catenin using an antibody against the NF-κB subunit p65. β-actin was used as a cytosolic marker, and fibrillarin served as a nuclear marker. [file 8373715.f1.docx]
